# A Randomized Controlled Trial Comparing the Efficacy of High-Frequency rTMS and Intermittent Theta-Burst Stimulation on Depressive and Anxiety Symptoms in Depressive Disorder

**DOI:** 10.1192/j.eurpsy.2025.761

**Published:** 2025-08-26

**Authors:** J. Opelka, J. Albrecht, O. Bezdíček

**Affiliations:** 1 Department of Neurology and Centre of Clinical Neuroscience, First Faculty of Medicine and General University Hospital in Prague, Charles University, Prague; 2 Department of Psychiatry, Most Hospital, Krajská zdravotní a.s., Most; 3 Psychiatric Clinic of the Faculty of Health Studies, Jan Evangelista Purkyně University and Masaryk Hospital in Ústí nad Labem, Krajská zdravotní a.s., Ústí nad Labem, Czech Republic

## Abstract

**Introduction:**

Depressive disorder is one of the most prevalent neuropsychiatric conditions in the world, significantly affecting both individuals and society. Despite numerous therapeutic options, many patients do not respond adequately to treatment, highlighting the need for novel approaches, in the case of this study repetitive transcranial magnetic stimulation (rTMS).

**Objectives:**

The main objective of this study was to compare the therapeutic efficacy of high-frequency rTMS (HF-rTMS) and intermittent theta-burst stimulation (iTBS) in reducing depressive and anxiety symptoms in patients with depressive disorder.

**Methods:**

This double-blind, randomized controlled trial was conducted at the psychiatric ward of Most Hospital. Patients (N=97) diagnosed with depressive disorder were randomly assigned to receive either HF-rTMS or iTBS both aimed at left dorsolateral prefrontal cortex. Data were collected using both self-assessment and clinician-rated questionnaires, such as the Zung Self-Rating Depression Scale (ZSDS), Beck Anxiety Inventory (BAI), Hamilton Depression Rating Scale (HAMD), and Hamilton Anxiety Rating Scale (HAMA), before and after 10 stimulation sessions.

**Results:**

The analysis showed a significant reduction in depressive and anxiety symptoms after ten stimulation sessions using both HF-rTMS and iTBS across all applied questionnaires. Specifically, the ANOVA results for the ZSDS demonstrated a significant decrease in symptoms over time (F=414, p<.001), with a mean reduction of 6.54 points (95% CI=4.64–8.43). Similarly, the HAMD scores showed a significant reduction (F=299.72, p<.001), with a mean reduction of 7.83 points (95% CI=5.79–9.87). For anxiety symptoms, the BAI revealed a significant decrease (F=389.26, p<.001), with a mean reduction of 5.72 points (95% CI=4.45–6.99) and the HAMA showed a similar trend (F=656.15, p<.001), with a mean reduction of 7.39 points (95% CI=5.58–9.20).

No significant difference in efficacy was found between the two stimulation protocols across all measures: ZSDS (F=0.142, p=0.237), HAMD (F=0.431, p=0.376), BAI (F=0.269, p=0.365), and HAMA (F=0.813, p=0.370).

**Conclusions:**

This study confirms that both HF-rTMS and iTBS are effective in reducing depressive and anxiety symptoms, with no significant difference in their efficacy across all measured outcomes after ten stimulations. However, iTBS offers distinct advantages over HF-rTMS, including a shorter stimulation duration and a lower incidence of side effects.

This study was supported by an Internal grant agency: IGA-KZ-2022-1-4 (417119001).

**Disclosure of Interest:**

None Declared

